# A Dual-Mean Statistical and Multivariate Framework for Machinability Evaluation in CNC Turning: Gradient and Stiffness Analysis Across Five Materials

**DOI:** 10.3390/ma18132952

**Published:** 2025-06-22

**Authors:** Mohammad S. Alsoufi

**Affiliations:** Department of Mechanical Engineering, College of Engineering and Architecture, Umm Al-Qura University, Makkah 21955, Saudi Arabia; mssoufi@uqu.edu.sa

**Keywords:** CNC turning, machinability, cutting force, deformation, efficiency, stiffness, PCA

## Abstract

This study proposes a dual-statistical and gradient-based framework to evaluate the machinability of five engineering alloys under CNC turning. Cutting force and surface deformation were measured across five machining zones. Finite difference-based gradients revealed spatial variations in material response. Stainless Steel 304 showed the highest cutting force (328 N), while Aluminum 6061 had the highest deformation (0.0164 mm). Carbon Steel 1020 exhibited the highest force-to-deformation efficiency (>97,000 N/mm). Arithmetic and harmonic means highlighted statistical sensitivities, while principal component analysis (PCA) identified clustering among materials and reduced dimensionality. A composite machinability score, integrating stiffness variation, efficiency gradients, and multivariate features, ranked Aluminum 6061 highest, followed by Brass C26000 and Bronze C51000. This methodology enables interpretable benchmarking and informed material selection in precision manufacturing.

## 1. Introduction

In contemporary high-precision manufacturing, the emphasis on material efficiency [1], surface integrity [2], and dimensional stability [3] has reintroduced attention to the intricate concept of machinability. This term describes the relationships between tool–workpiece interaction, thermomechanical properties, and dynamic material response [4,5]. While cutting force [6] and surface roughness [7,8] have traditionally been used as indicators of machinability, they are frequently assessed separately, overlooking the essential energy mechanisms and material stiffness characteristics that change throughout the cutting path [9]. This issue is particularly relevant in computer numerical control (CNC) turning operations, where tool overhang, clamping stiffness, and thermal dissipation alter progressively along axial cutting zones. This leads to varied stress–strain fields and localized thermal softening effects [10,11,12]. Comprehending these complexities is crucial for refining machining processes, as it enables the formulation of more efficient cutting strategies that can extend tool life and enhance overall part quality [13].

Recent advancements in machining diagnostics have shifted toward integrating direct measurements, such as cutting force (*F*_c_) and surface deformation (*δ*), with derived indicators, including force gradients, stiffness coefficients, and efficiency ratios [14,15]. These metrics offer a deeper understanding of material resistance to shear, its ability to undergo plastic deformation under thermomechanical loads, and the overall energy expenditure of the cutting process [16]. Nevertheless, existing machinability frameworks often lack statistical rigor. Arithmetic means are inadequate for handling non-linear or skewed distributions, especially in zone-based studies where machining behavior fluctuates dynamically along the toolpath [17,18].

In this context, both arithmetic and harmonic means have shown significant statistical advantages for assessing multiplicative or reciprocal relationships in various engineering fields [19,20]. However, their use in machining science is still uncommon. The harmonic mean is particularly effective for evaluating force-to-deformation efficiency and stiffness, focusing on reciprocal metrics. Conversely, the arithmetic mean offers a balanced assessment that accounts for both proportional relationships and data variation, minimizing the impact of outliers. Thus, a dual-mean framework can uncover hidden sensitivities in derived metrics like force gradient ($\frac{dF}{dx}$), deformation gradient ($\frac{d\delta }{dx}$), and stiffness ($\frac{dF}{d\delta }$), which are vital for evaluating material performance variability during machining processes [21,22].

Although the literature examines force modeling and strain responses in machining, few studies have implemented a spatially distributed experimental approach that identifies zone-specific machinability gradients [23,24]. Even fewer have combined this with various statistical treatments and multi-parametric analysis methods. The use of multivariate tools like principal component analysis (PCA) in machining remains limited, despite their established effectiveness in reducing dimensionality and assessing feature interdependence in materials science and structural mechanics [25,26]. Moreover, the lack of a unified machinability index that integrates both primary and derived parameters within spatial and statistical domains limits the ability to perform effective comparative benchmarking across different material types. Recent studies have demonstrated the effectiveness of principal component analysis (PCA) in machining research for dimensionality reduction, process monitoring, and interpreting multivariable machining responses. For example, Joshi et al. [27] applied principal component analysis (PCA) to analyze cutting forces and monitor the progression of tool wear during CNC turning. Lin et al. [28] utilized PCA to extract dominant features from force signals in high-speed machining, whereas Kozłowski et al. [29] employed PCA to correlate surface roughness with cutting conditions and tool states. These studies confirm the diagnostic potential of PCA in capturing latent machining features. Yet, prior work has focused mainly on tool wear or surface roughness, often neglecting stiffness gradients and deformation trends across machining zones.

To address the methodological and analytical gaps, this study presents a statistically revised, multi-zonal evaluation of machining performance for five key engineering materials: Aluminum Alloy 6061, Brass C26000, Bronze C51000, Stainless Steel 304 (annealed), and Carbon Steel 1020 (annealed). These materials encompass a broad range of thermal conductivity, yield strength, and ductility, making them an excellent testbed for comparative machinability assessment. With a CNC turning setup, cutting force and surface deformation were recorded at five evenly spaced zones along the machined length, facilitating the evaluation of tool engagement impacts, thermal gradients, and material compliance under practical machining conditions.

The raw data were processed through both arithmetic and harmonic means, allowing for the calculation of average behavior as well as derived metrics like force-to-deformation efficiency, stiffness, and their gradients. Next, a PCA-based clustering analysis was employed to condense multidimensional machining data into distinct clusters, thereby enhancing the understanding of how material properties influence process responses. A composite machinability score was developed to synthesize these diverse metrics into a single, evaluative measure that reflects both energy consumption and mechanical responsiveness, enabling an objective comparison across materials.

The present study introduces a novel framework for evaluating machinability that integrates zone-based measurement, dual-statistical analysis, multivariate data compression, and composite scoring. This approach is highly scalable, adaptable to various machining operations, and provides essential insights for tool design, optimizing parameters, and selecting materials in high-performance manufacturing settings [30,31,32].

This study introduces a dual-statistical framework that utilizes both arithmetic and harmonic means to effectively characterize cutting force, deformation, force-to-deformation efficiency, stiffness gradients, and multivariate machining behavior through PCA. It expands upon the authors’ previous research on surface integrity and measurement accuracy in CNC turning processes [11,12,33], broadening the analysis framework to focus on gradient-based characterization of machinability. The proposed framework enables thorough benchmarking of material machinability in a controlled CNC turning environment.

## 2. Materials and Experimental Methods

### 2.1. Workpiece Materials

**Figure 1:**
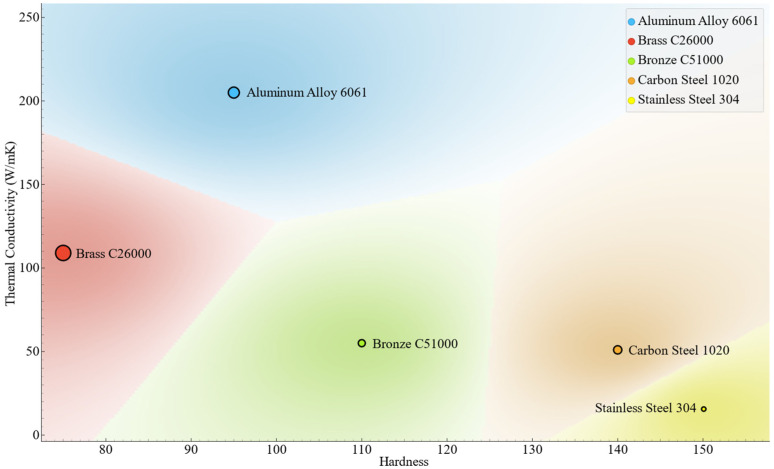
Hardness vs. thermal conductivity for the five tested engineering materials, with colored regions indicating material clusters and the size of points indicating the machinability index.

Five distinct engineering materials were chosen to demonstrate a broad array of mechanical and thermal properties commonly observed in industrial machining: Aluminum Alloy 6061, Brass C26000, Bronze C51000, Stainless Steel 304 Annealed, and Carbon Steel 1020 Annealed (SpecLine Arabia Company Ltd. and Al Mokahal Co., Al-Jubail, Saudi Arabia). These materials were selected for their varied hardness, thermal conductivity, and structural characteristics, facilitating a comparative analysis of their machinability during controlled turning operations, as shown in Figure 1.

Aluminum Alloy 6061 is a lightweight, ductile material recognized for its high thermal conductivity (approximately 205 W/m·K) and moderate hardness (around 95 HB). It is often selected for applications demanding superior corrosion resistance and effective thermal management. However, its reduced hardness may result in the formation of built-up edge (BUE) and smearing during machining, potentially impacting surface integrity.

Brass C26000 is an alloy consisting of copper and zinc, known for its excellent machinability, moderate strength, and medium thermal conductivity (around 109 W/m·K). It features lower cutting resistance and consistent chip formation, making it an ideal standard for evaluating machinability.

Bronze C51000, crafted from a tin bronze alloy, delivers outstanding wear resistance and robust mechanical strength. Its well-balanced hardness and thermal conductivity contribute to maintaining dimensional stability during machining, although they may result in moderate tool wear, depending on the cutting speeds and feed rates.

Stainless Steel 304 Annealed is an austenitic stainless steel known for its low thermal conductivity (around 16 W/m·K), excellent ductility, and significant work hardening ability. While it provides impressive corrosion resistance and structural integrity, machining 304 stainless steel can be challenging due to the formation of built-up edges (BUE), increased cutting temperatures, and considerable tool wear rates.

Carbon Steel 1020 Annealed is a low-carbon steel known for its moderate strength and excellent machinability. It strikes a balance of hardness (about 140 HB), thermal conductivity (approximately 51 W/m·K), and ductility. The annealed condition improves machinability by reducing hardness and enhancing chip formation characteristics.

All materials were prepared as cylindrical bar stocks, guaranteeing consistent diameters and lengths for uniform experimental conditions. Prior to machining, the mechanical properties were verified through Brinell hardness testing, and thermal conductivity values were obtained from reputable literature to ensure consistency. These materials provided a robust foundation for examining cutting force, deformation, and efficiency metrics across various cutting zones.

To further support the interpretation of the experimental results, Table 1 summarizes the key mechanical and thermal properties of the five investigated materials, including yield stress, ultimate tensile strength, hardness, thermal conductivity, density, and Young’s modulus (also known as the elastic modulus). These parameters complement the hardness and thermal conductivity values already discussed, enhancing the understanding of each material’s machining behavior.

Machinability classifications and machining recommendations for the selected materials were referenced against ISO 513 guidelines [34]. Tooling selection, tool wear criteria, and cutting condition recommendations were based on ISO 3685 standards [35]. This ensures that the experimental setup aligns with internationally accepted practices for machinability evaluation. The chemical and physical properties summarized in Table 1 are consistent with values reported in the ASM Handbooks [36], the MatWeb database [37], and verified manufacturer data [38], providing a reliable foundation for interpreting material behavior during CNC turning.

### 2.2. CNC Turning Setup

Machining experiments were conducted using a high-precision Gate-Eclipse ECL-400 CNC lathe (Gate Machinery International Ltd., Derbyshire, UK), equipped with a Fagor CNC control system to ensure precise management of cutting parameters. The machine’s rigidity and thermal stability helped maintain consistent machining conditions. All experiments were conducted in a laboratory where temperature and humidity were controlled at 22 ± 1 °C and 50 ± 5% relative humidity, respectively, to minimize environmental influences.

The cylindrical workpieces were securely held in a rigid three-jaw chuck, guaranteeing accurate axial alignment and minimizing vibration during cutting. During the trials, dry-cutting conditions were consistently applied to assess the impact of the material’s thermal properties without the influence of lubricants. All tests maintained a uniform depth of cut of 0.25 mm, with cutting speed and feed rate adjusted based on the physical properties of the material.

Aluminum Alloy 6061 was machined at a cutting speed of 120 m/min with feed rates ranging from 0.25 to 0.05 mm/rev. Bronze C51000 was processed at 60 m/min using the same feed rates. Brass C26000, Stainless Steel 304 Annealed, and Carbon Steel 1020 Annealed were machined at a reduced speed of 30 m/min with finer feed rates ranging from 0.125 to 0.025 mm/rev. Initial diameters were 28.5 mm for Aluminum 6061, 31.5 mm for Brass C26000, and 34.0 mm for Bronze C51000. The total machined length of 45 mm was divided into five axial zones, each 8.7 mm long, with center positions at 5.85, 14.55, 23.25, 31.95, and 40.65 mm from the chuck, allowing for spatially resolved analysis.

TiAlN-coated tungsten carbide inserts (ISO CNMG 120408-PM [39]) were used due to their excellent wear resistance and heat tolerance. Inserts were replaced after every five passes or sooner if flank wear exceeded 0.3 mm, following ISO 3685 guidelines [35]. The cutting inserts used had a rake angle of 12° and a clearance angle of 7°, selected to ensure balanced cutting performance and tool life across all tested materials.

Key components of the experimental setup, including the CNC turning system and dimensional measurement tools, follow configurations previously described in our prior works [33,34,35], and the present paper provides a detailed account of the force and deformation measurement methodology. The force and deformation measurement system is described in detail in the next section.

### 2.3. Measurement Techniques

The assessment of machinability was based on real-time monitoring of the primary cutting force (*F*_c_) and corresponding surface deformation (*δ*) using calibrated, high-resolution sensors.

Cutting forces were measured using a TeLc DKM2010 three-component turning dynamometer (TeLc GmbH, Bensheim, Germany), capable of resolving up to 2000 N with a resolution of 1 N and 0.01% accuracy. The dynamometer, installed beneath the tool holder, recorded orthogonal force components via strain gauge technology. A high-speed acquisition system allowed adjustable sampling rates between 5 and 100 Hz. For this study, only the primary cutting force (*F*_c_) was analyzed, given its dominance in representing material resistance to shear.

Deformation measurements were obtained using a Keyence LK-G5000 laser displacement sensor (Keyence Corporation, Osaka, Japan), configured in a stationary, non-contact arrangement. The system provided micrometer-scale precision and fast temporal response, enabling continuous tracking of elastic–plastic surface deflection as the tool interacted with the material.

Both systems were calibrated using certified standards prior to data acquisition. A digital Butterworth low-pass filter (cutoff at 10 Hz) was applied to eliminate high-frequency noise and isolate meaningful force and deformation signals.

Each experiment was repeated three times under identical conditions, and the resulting data were averaged. Both arithmetic and harmonic means were used to characterize central tendencies to enhance robustness. These dual-mean values formed the basis for computing derived metrics such as the cutting force gradient ($\frac{dF}{dx}$), deformation gradient ($\frac{d\delta }{dx}$), and stiffness ($\frac{dF}{d\delta }$). This approach facilitated deeper insights into the dynamic behavior of each material during CNC turning and supported the development of composite machinability indicators.

## 3. Statistical and Analytical Methods

### 3.1. Arithmetic and Harmonic Mean Calculations

This study employs arithmetic and harmonic mean methods to analyze machining reactions across various materials. It analyzes cutting force and deformation data collected from five machining zones. These statistical methods provide a dual-perspective analysis of machining behavior, incorporating diverse material responses and minimizing distortion from data variability or outliers. The arithmetic mean (AM) is defined as follows:

(1) $$\mathrm{AM}=\frac{1}{n}\overset{n}{\underset{i=1}{\sum }}x_{i}$$

This metric is especially appropriate when the data exhibit additive relationships or all values contribute equally to the total magnitude, as observed with cutting forces influenced by cumulative material resistance and tool engagement effects. It provides a central tendency that reflects the overall average. However, extreme outliers can strongly influence it, making it more representative of total load accumulation or energy input over time. Consequently, the arithmetic mean is well-suited for analyzing materials where the cutting force increases steadily or irregularly due to progressive tool wear, temperature rise, or material heterogeneity. In contrast, the harmonic mean (HM) is defined as

(2) $$\mathrm{HM}=\frac{n}{\sum_{i=1}^{n}\frac{1}{x_{i}}}$$

This approach inherently emphasizes smaller values and is particularly suitable for metrics expressed as rates, such as force per unit deformation or energy per unit length. It is especially valuable in contexts where minimizing the impact of extreme outliers is critical to preserving analytical robustness. In the present study, the harmonic mean offers a conservative yet stable representation of machining behavior. It is well-suited for characterizing materials that exhibit consistent mechanical compliance and low variability across machining zones.

The utilization of both statistical methodologies facilitates a comprehensive and corroborated examination of the machining process. The arithmetic mean frequently highlights substantial trends, such as peak cutting forces or regions of considerable deformation, but it may conceal more subtle or persistent trends present in softer materials. Conversely, the harmonic mean tends to mitigate abrupt fluctuations, offering a clearer perspective on baseline material behavior, although it may underestimate transient mechanical surges.

In this investigation, AM and HM were individually applied to the raw cutting force and deformation data from the five machining zones of each material. This dual analysis ensures a comprehensive evaluation of both energy demand (force-driven) and compliance behavior (deformation-driven), facilitating a more thorough comparison of machinability profiles among the chosen materials.

It should be noted that polynomial fitting in this study was applied primarily to visualize the smooth progression of cutting force and deformation across the predefined machining zones and not for predictive modeling. The high *R*^2^ values obtained reflect the well-structured, monotonic nature of the machining response along the toolpath and the controlled experimental design, rather than an absence of measurement variability.

### 3.2. Gradient Analysis

To capture the dynamic response of materials along the toolpath, this study applied numerical gradient analysis to both cutting force and deformation profiles. The experimental procedure employed a constant feed rate of 0.1 mm/rev and a constant depth of cut of 0.2 mm across all machining zones and materials. The spindle speed was maintained at 600 rpm, corresponding to cutting speeds appropriate for the tested materials per ISO 3685 guidelines [35]. Each machining zone was processed and measured in three independent repetitions to ensure statistical robustness. The results presented in this study represent the average of these three repetitions for each zone and material. This approach ensures that the derived gradients and stiffness trends are both statistically valid and representative of true machining behavior. In this experimental design, the machining zone index served as a controlled variable representing progressive tool engagement along the axial direction. This allowed systematic observation of how force, deformation, stiffness, and related responses evolved as a function of machining progression, capturing the cumulative effects of tool wear, thermal gradients, strain hardening, and material response under consistent cutting conditions.

Gradients offered a first-order approximation of how rapidly these quantities changed as a function of machining progression along the toolpath (i.e., across the defined machining zones), enabling localized assessment of material behavior under load. Specifically, the gradients of cutting force and deformation with respect to axial position, denoted as $\frac{dF}{dx}$ and $\frac{d\delta }{dx}$, respectively, were computed using finite difference methods over five discrete machining zones.

(3) $$\frac{dF}{dx}\approx \frac{F_{i+1}-F_{i}}{x_{i+1}-x_{i}}$$

(4) $$\frac{d\delta }{dx}\approx \frac{\delta_{i+1}-\delta_{i}}{x_{i+1}-x_{i}}$$

Here, $x_{i}$ represents the axial location of the center of each machining zone, while $F_{i}$ and $\delta_{i}$ are the corresponding cutting force and deformation measurements. These gradients were evaluated separately for both arithmetic and harmonic mean datasets, ensuring consistency with the dual-statistical framework established in Section 3.1.

It should be noted that since this analysis is based on discrete zone-wise data, gradients were computed using finite difference approximations rather than continuous differentiation.

The cutting force gradient ($\frac{dF}{dx}$) serves as a proxy for the rate at which mechanical energy input escalates along the cutting path. High positive gradients typically indicate material hardening, increased tool engagement, or the onset of unstable cutting conditions, especially in materials with significant strain rate sensitivity or structural heterogeneity. Conversely, low or negative gradients may reflect improved chip evacuation, thermal softening, or transitional effects near the free end of the workpiece.

The deformation gradient ($\frac{d\delta }{dx}$) illustrates how material compliance changes when subjected to a load. Steeper gradients imply localized softening, insufficient material support, or thermal expansion. In contrast, a flatter gradient signifies more uniform deformation, typically linked to materials that demonstrate stable mechanical properties or robust structural rigidity while cutting. By coupling these two gradients, the study also derived the material stiffness (*K*), defined as the ratio of the cutting force gradient to the deformation gradient across the toolpath, and it can be expressed as follows:

(5) $$K=\frac{dF}{d\delta }=\frac{\frac{dF}{dx}}{\frac{d\delta }{dx}}.$$

This formulation facilitates the evaluation of zone-specific stiffness through the force-to-deformation rate, enhancing the precision in detecting variations in rigidity throughout the cutting trajectory. Unlike conventional stiffness metrics that depend on bulk properties, this gradient-based method accurately reflects the mechanical response throughout processing by considering the effect of different variables such as temperature elevation, strain hardening, tool degradation, and material anisotropy.

The gradient analysis framework detects local transitions in machinability behavior while bridging raw measurements with meaningful indicators, especially when considered alongside mean-based metrics and multivariate tools like PCA (see Section 4.4).

It is acknowledged that machining with a small depth of cut and moderate feed rate can potentially result in thread-like surface roughness patterns due to feed marks. In this study, surface inspections were conducted to verify that such patterns remained within acceptable roughness limits and did not compromise the accuracy of force or deformation measurements. The selected machining parameters were carefully chosen to optimize sensitivity for gradient analysis while maintaining surface integrity, in accordance with recommendations from ISO 3685 [35].

### 3.3. Efficiency and Stiffness Metrics

This study provides two derived metrics, force-to-deformation efficiency and in-process stiffness, to evaluate the energetic demand and mechanical resistance experienced during cutting, complementing the gradient-based evaluation of machining response. These metrics enhance interpretive depth by correlating force and deformation measurements with physically significant formulations.

The force-to-deformation efficiency, denoted as *η*, is defined as the ratio of cutting force to deformation for each machining zone:

(6) $$\eta =\frac{F}{\delta }$$

This metric quantifies the force used to achieve unit deformation and indicates the energetic expense of material displacement. A high efficiency number indicates that the material exhibits significant resistance to deformation and requires increased energy for material removal. This behavior is generally linked to elevated yield strength, enhanced strain-hardening capability, or microstructural resilience. In contrast, low efficiency numbers indicate a material that deforms more easily under minimal effort, generally associated with ductility, thermal softening, or diminished material integrity under cutting-induced stress.

In contrast to stiffness, which assesses the rate of change between force and deformation, the efficiency metric highlights their proportionate relationship. It is especially beneficial for steady-state comparisons among materials, since it encapsulates the overall resistance behavior, which helps eliminate the disturbances caused by point-wise fluctuations. The efficiency was calculated using both arithmetic and harmonic means. The arithmetic mean highlights elevated force or deformation instances and is consequently more responsive to peak energy requirements. The harmonic mean offers a refined representation that diminishes the influence of large values, emphasizing baseline behavior and mitigating transient spikes caused by tool vibration, chip adhesion, or local instability.

The in-process stiffness, previously defined in Equation (5), is revisited here to emphasize its importance in capturing real-time mechanical resistance during cutting. Unlike bulk stiffness metrics such as Young’s modulus, this localized gradient reflects the evolving interplay between cutting force and deformation throughout the toolpath. It accounts for process-dependent phenomena like strain hardening, thermal expansion, and tool wear. When analyzed using both arithmetic and harmonic mean datasets, this metric reveals not only abrupt stiffness fluctuations in rigid materials but also steady-state compliance in ductile alloys. Thus, in-process stiffness serves as a dynamic, geometry-aware indicator of machining stability and energy transfer.

The efficiency of force-to-deformation and in-process stiffness collectively establish a dual framework for analyzing energy input and material resistance during actual machining circumstances. These measures enhance the comprehension of material behavior beyond mere force or deformation trends and provide a more rigorous machinability comparison among various technical alloys.

### 3.4. Principal Component Analysis (PCA)

To reduce the dimensionality of the derived machining metrics and identify the underlying structure in the multivariate dataset, principal component analysis (PCA) was employed. PCA is an unsupervised statistical technique that transforms a set of possibly correlated variables into linearly uncorrelated variables called principal components. These components are constructed such that the first principal component (PC1) captures the maximum variance present in the data, followed by PC2, and so forth, under the constraint of mutual orthogonality.

PCA was performed on the matrix of machining features derived independently from both arithmetic and harmonic mean datasets. The feature vector included five key variables per material: average cutting force, average deformation, force gradient ($\frac{dF}{dx}$), deformation gradient ($\frac{d\delta }{dx}$), and in-process stiffness ($\frac{dF}{d\delta }$). All features were first standardized to zero mean and unit variance prior to PCA application to eliminate scale dominance and ensure uniform influence across variables. The linear transformation of the original feature space is defined by the eigenvectors of the covariance matrix Σ, where

(7) $$\sum =\frac{1}{n-1}X^{T}X$$

and the principal components are given by

(8) Z=X·W

Here, *X* is the standardized data matrix, *W* is the matrix of eigenvectors (principal directions), and *Z* is the projected data in the principal component space. The corresponding eigenvalues indicate the proportion of variance explained by each component.

The PCA was performed independently on arithmetic and harmonic datasets to examine the impact of mean selection on feature weighting and material clustering. Notwithstanding the numerical discrepancies in mean treatment, all PCA methods settled on two principal components that together accounted for approximately 95% of the total variation. PC1 was predominantly linked to stiffness-related variables and the degree of cutting force, while PC2 was more affected by deformation behavior and gradient fluctuations.

This dimensionality reduction allowed for visually clustering materials in PC1–PC2 space, enhancing the intuitive understanding of machining features. Materials exhibiting analogous machining characteristics, such as Brass C26000 and Bronze C51000, were tightly grouped, while dissimilar materials like Aluminum Alloy 6061 and Stainless Steel 304 Annealed occupied discrete areas owing to their distinctive mixes of compliance, stiffness, and energy requirements.

The PCA results elucidated the relative contributions of each machining characteristic. High PC1 loadings on stiffness and force gradient indicate that mechanically resistant materials predominantly influenced this axis. Conversely, materials exhibiting elevated deformation gradients and diminished force-to-deformation efficiency were represented along PC2, indicating compliant and ductile characteristics.

PCA reduced the intricate interrelationships among force, deformation, and derived metrics while enhancing the robustness of the dual-mean framework. It facilitated uniform material classification from both statistical viewpoints and established the foundation for ranking materials based on composite machinability scores, as described in the following section.

### 3.5. Composite Machinability Score

A composite machinability score (*CMS*) was developed for each material to consolidate the multifaceted machining behavior into a singular, interpretable metric, utilizing the normalized contributions of essential mechanical and energetic indications. This index functions as a comprehensive performance indicator that facilitates quantitative material machinability comparisons through arithmetic and harmonic mean analysis.

The development of the *CMS* was grounded in five principal machining metrics: mean cutting force (*F*), mean deformation (*δ*), force gradient $(\frac{dF}{dx}$), deformation gradient ($\frac{d\delta }{dx}$), and in-process stiffness ($\frac{dF}{d\delta }$). Each metric was computed individually for all materials across the machining zones using both arithmetic and harmonic formulations. To ensure uniform comparability, all variables were min–max normalized onto a scale from 0 to 1 using the expression

(9) $$X_{norm}=\frac{X-X_{min}}{X_{max}-X_{min}},$$

where *X* is the raw value of the metric for a given material, while *X_min_* and *X_max_* represent the minimum and maximum values of the metric *X* across all materials, respectively. The term $X_{ij}^{*}$ is introduced to represent the normalized value of the *i*-th machinability metric for the *j*-th material, and it can be obtained by applying Equation (9) to each individual metric across all materials.

For metrics where higher values indicate improved machinability, the corresponding normalized term $X_{ij}^{*}$ increases proportionally. In contrast, for metrics where higher values imply lower machinability (e.g., cutting force, deformation, and stiffness), the complement of the normalized value ($1-X_{ij}^{*}$) was used to preserve a consistent interpretation, namely, that higher normalized values indicate better machinability across all metrics.

The composite machinability score $CMS_{j}$ for each material *j* was calculated as the average of its normalized scores, including inverted ones where applicable.

(10) $$CMS_{j}=\frac{1}{n}\overset{n}{\underset{i=1}{\sum }}{\tilde{X}}_{ij}$$

where ${\tilde{X}}_{ij}=X_{ij}^{*}$ if higher values of the metric *i* correlate with improved machinability, and ${\tilde{X}}_{ij}=1-X_{ij}^{*}$ if lower values of the metric *i* correlate with improved machinability. Furthermore, *n* = 5 represents the total number of metrics considered. This method of unweighted averaging ensures a balanced representation of all machinability dimensions, avoiding undue emphasis on any single factor unless weighting is explicitly applied.

The *CMS* values were computed independently for the arithmetic and harmonic mean datasets, enabling dual benchmarking. Materials with elevated *CMS* scores indicate superior machinability, characterized by diminished cutting force, uniform and moderate deformation, minimum gradient fluctuations, and consistent stiffness during processing. Conversely, reduced *CMS* values indicate that the materials necessitate increased energy for machining, exhibit irregular deformation patterns, or undergo abrupt variations in mechanical resistance during tool movement.

This composite index improves the multivariate framework established in PCA, enabling a distinct ranking of machinability among the assessed materials. The correlation between PCA clustering and *CMS* rankings validates the internal consistency of the dual-mean analytical method, affirming the reliability of the selected machining measures as performance differentiators.

## 4. Results and Discussions

### 4.1. Force and Deformation Responses Across Cutting Zones

**Figure 2:**
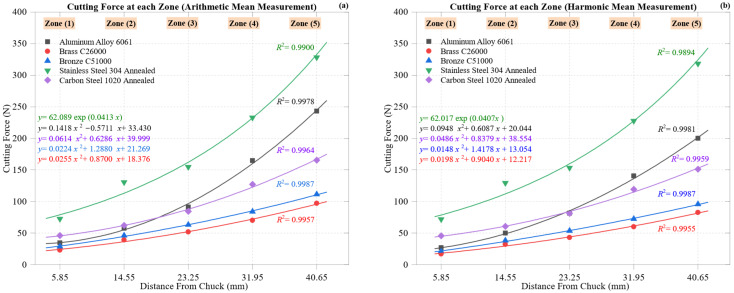
Cutting force distribution across machining zones; (**a**) AM and (**b**) HM approaches.

Figure 2 presents the cutting force distribution across five machining zones for the five studied materials, based on arithmetic mean (Figure 2a) and harmonic mean (Figure 2b) values. Across both formulations, cutting force increases steadily from Zone 1 (5.85 mm from the chuck) to Zone 5 (40.65 mm), reflecting a consistent escalation in machining resistance due to accumulated tool wear, thermal loading, and reduced support near the free end.

Stainless Steel 304 Annealed exhibits the steepest force escalation, rising from ~73 N at Zone 1 to 328 N (AM) and 318 N (HM) at Zone 5. This non-linear rise reflects the material’s low thermal conductivity and substantial strain hardening, elevating tool–workpiece friction. The fits are robust, with *R*^2^ values of 0.9900 (AM) and 0.9894 (HM).

Carbon Steel 1020 Annealed shows a moderate increase of 46 N to 165 N (AM) and 46 N to 151 N (HM), consistent with its ferritic–pearlitic microstructure, which offers moderate compliance. Fitting accuracy remains high, with *R*^2^ values of 0.9964 and 0.9959.

Bronze C51000 maintains a mid-range profile of 29 N to 111 N (AM) and 21 N to 96 N (HM), consistent with its moderate hardness and thermal conductivity. The trends are smooth, with *R*^2^ value of 0.9987 in both cases.

Aluminum Alloy 6061, the most thermally conductive and ductile, exhibits the most gradual force rise: from 34.6 N to 243 N (AM) and 27.0 N to 200 N (HM). The curved progression, particularly in the AM, suggests built-up edge (BUE) or thermal distortion effects, consistent with prior studies [40,41]. High fit reliability (*R*^2^ = 0.9978 and 0.9981) supports this trend.

Brass C26000 exhibits the most linear and stable force response, ranging from 23.1 N to 97.0 N (AM) and 17.0 N to 82.5 N (HM), indicating excellent machinability. The nearly linear trend yields strong fits, with *R*^2^ values of 0.9957 and 0.9955.

Material rankings remain consistent across both means. However, harmonic mean curves exhibit lower peaks and a smoother progression, especially in high-force materials, due to their tendency to mitigate outliers such as chip adhesion or chatter.

Together, both methods provide complementary insights; the AM highlights energy-intensive peaks, while the HM smooths noise and reveals baseline trends, offering a dual perspective on the evolution of machinability.

**Figure 3:**
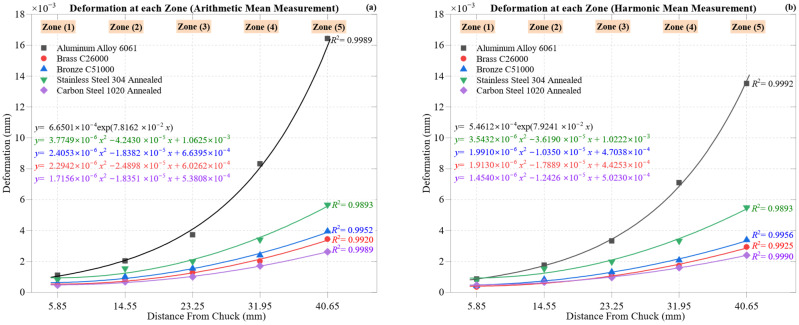
Deformation distribution across machining zones; (**a**) AM and (**b**) HM approaches.

Figure 3 illustrates the progression of maximum deformation across five machining zones for all five materials using arithmetic mean (Figure 3a) and harmonic mean (Figure 3b) formulations. Both plots reveal a consistent upward trend in deformation from Zone 1 (5.85 mm) to Zone 5 (40.65 mm), reflecting tool wear, thermal softening, reduced structural support, and increased cutting force. However, the degree and rate of deformation vary by material, as do the shapes of the curves.

Aluminum Alloy 6061 exhibits the highest deformation, characterized by a unique exponential growth pattern. It increases from 0.0011 mm to 0.0164 mm (AM), while the harmonic mean moderates the peak to 0.0135 mm, illustrating the HM’s attenuation of outlier values. The exponential fits are strong (*R*^2^ = 0.9989 [AM] and 0.9992 [HM]), underscoring the influence of thermal softening, ductility, and BUE formation.

Carbon Steel 1020 Annealed exhibits the lowest deformation, rising from 0.00047 mm to 0.00263 mm (AM) and 0.00240 mm (HM), with nearly linear behavior (*R*^2^ = 0.9989 and 0.9990), reflecting its stiffness and elastic stability.

Brass C26000 and Bronze C51000 occupy the mid-range. The brass increases from 0.00047 mm to 0.00345 mm (AM) and 0.00293 mm (HM); the bronze from 0.00058 mm to 0.00395 mm (AM) and 0.00339 mm (HM). Both show strong fits: the brass *R*^2^ = 0.9920/0.9925 and the bronze *R*^2^ = 0.9952/0.9956, indicating stable compliance.

Stainless Steel 304 Annealed displays moderate deformation despite high cutting forces, ranging from 0.00081 mm to 0.00567 mm (AM) and 0.00550 mm (HM). Both curves fit well (*R*^2^ = 0.9893), indicating a gradual accumulation of strain under increasing load and temperature.

While material rankings remain unchanged, harmonic mean curves consistently reduce peak values in ductile materials, such as the aluminum and bronze, confirming their ability to suppress local spikes due to chatter or chip adhesion.

All materials exhibit minimum deformation near the chuck (Zone 1) and peak values at Zone 5, due to loss of support and cumulative thermal effects toward the workpiece end.

Together, AM and HM formulations provide a comprehensive view of deformation behavior, validating the data’s robustness and forming a foundation for the efficiency and stiffness analysis that follows.

### 4.2. Variability and Gradient Analysis

Statistical visualization using boxplots enriched with notched boxes, whiskers, and violin overlays offers a comprehensive framework for evaluating cutting force variability. Notched boxes represent the interquartile range (IQR) and median, whiskers identify typical fluctuations and outliers, and violin plots depict the full distribution shape. This layered visualization helps distinguish steady-state behavior from anomalous machining responses.

In this section, gradient analysis refers to evaluating the gradients of cutting force ($\frac{dF}{dx}$) and deformation ($\frac{d\delta }{dx}$) across zones. These finite difference-based metrics quantify how rapidly these responses change with tool progression, offering insights into material stability and load response.

**Figure 4:**
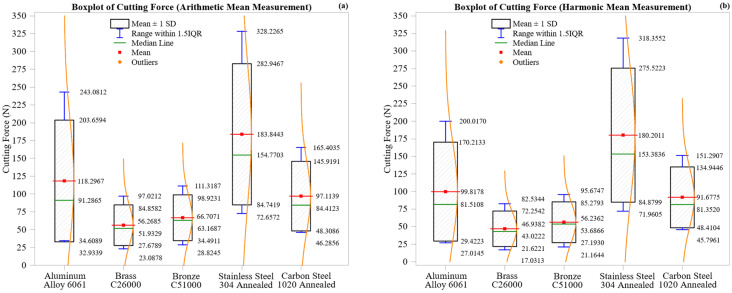
Boxplot analysis of cutting force; (**a**) AM and (**b**) HM measurements.

Figure 4 shows boxplots of cutting force values across materials using arithmetic mean (Figure 4a) and harmonic mean (Figure 4b) formulations.

Stainless Steel 304 Annealed shows the highest mean force and variability: 183.84 N (AM), 180.20 N (HM), with wide ± 1 SD and IQR ranges. This positively skewed profile reflects the material’s high strain hardening, poor thermal conductivity (~16 W/m·K), and progressive tool–material friction. Localized thermal softening and flank wear further explain the pronounced force asymmetry [42].

Aluminum Alloy 6061 displays a broad, skewed distribution: AM mean = 118.30 N, HM mean = 99.82 N, with long tails and lower median values. This highlights susceptibility to BUE formation, smearing, and thermal distortion, especially in dry cutting.

Carbon Steel 1020 Annealed shows balanced force behavior: 97.11 N (AM), 91.68 N (HM), with narrow, symmetric distributions. The material’s moderate rigidity and ferritic–pearlitic structure promote machining consistency.

Bronze C51000 presents mid-range values of 66.71 N (AM) and 56.24 N (HM), with slightly broader variability than brass. This is due to higher hardness and increased tool resistance.

Brass C26000, the most machinable material, exhibits the lowest force values and the tightest spread, with values of 56.27 N (AM) and 46.94 N (HM), a compact interquartile range (IQR), and low variability. This reflects excellent thermal conductivity, chip fragmentation, and minimal tool adhesion.

Across all materials, harmonic means consistently produce tighter, less skewed distributions, particularly in the aluminum and stainless steel, demonstrating their resilience to outliers and suitability for stability assessment. In contrast, arithmetic means highlight peak demands and process irregularities, providing insights into worst-case machining conditions.

This dual-statistical boxplot analysis clearly differentiates machining consistency across materials, affirming the value of combining arithmetic mean (AM) and harmonic mean (HM) for robust machinability assessment.

**Figure 5:**
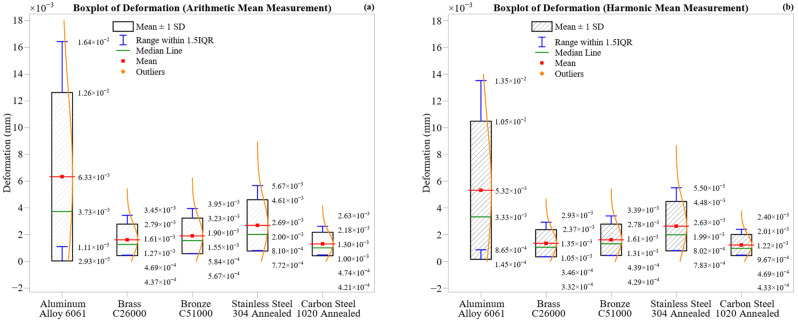
Boxplot analysis of deformation; (**a**) AM and (**b**) HM measurements.

Figure 5 illustrates the distribution of maximum deformation across all machining zones for the five materials, shown using boxplots and violin plots for both arithmetic mean (Figure 5a) and harmonic mean (Figure 5b) datasets. Each boxplot includes annotations for the mean (red), median (green), ±1 standard deviation (blue whiskers), and overlaid violin shapes that reflect the distribution density of the deformation, allowing for a comparative evaluation of magnitude, symmetry, and variability.

Aluminum Alloy 6061 exhibits the highest deformation and the widest spread. Its arithmetic mean is 0.00633 mm (median 0.00373 mm) with a broad ± 1 SD interval from 0.00111 mm to 0.01260 mm, and a 1.5 IQR range extending to 0.01643 mm. The harmonic mean shows slightly lower values of 0.00532 mm (mean) and 0.00333 mm (median), with a narrower spread. The pronounced skewness in both plots reflects exponential-like deformation growth due to thermal effects, ductility, and built-up edge (BUE) formation at longer cutting distances.

Carbon Steel 1020 Annealed exhibits the least deformation, with compact, symmetric distributions of 0.00130 mm (AM) and 0.00122 mm (HM), indicating tight ± 1 SD and IQR ranges. This confirms the steel’s rigidity and strong resistance to plastic deformation even under cumulative loading.

Brass C26000 and Bronze C51000 lie in the mid-range. The brass shows a mean of 0.00161 mm (AM) and 0.00135 mm (HM), while the bronze yields slightly higher values: 0.00190 mm (AM) and 0.00161 mm (HM). Both have moderate spreads and symmetric distributions, with the bronze’s slightly broader range reflecting its higher hardness and less ductile nature.

Stainless Steel 304 Annealed shows moderate deformation with relatively low variability. The arithmetic and harmonic means are close, with values of 0.00269 mm and 0.00263 mm, respectively, and nearly identical medians and narrow spreads. This reflects predictable strain accumulation due to strain hardening, despite the material initially exhibiting strong resistance to deformation.

Harmonic mean plots consistently reduce deformation magnitudes and suppress outliers, especially in the aluminum and bronze, while preserving the material rankings observed in AM data. In contrast, AM values remain more sensitive to local spikes, offering better insight into cumulative plastic strain in ductile materials.

As shown in Figure 5, Aluminum 6061 exhibits the most compliance under load, while Carbon Steel 1020 displays the maximum dimensional stability. The brass and bronze fall in between, exhibiting consistent and predictable behavior. The combined AM–HM analysis enhances interpretation by capturing both extreme and steady-state deformation behavior.

**Figure 6:**
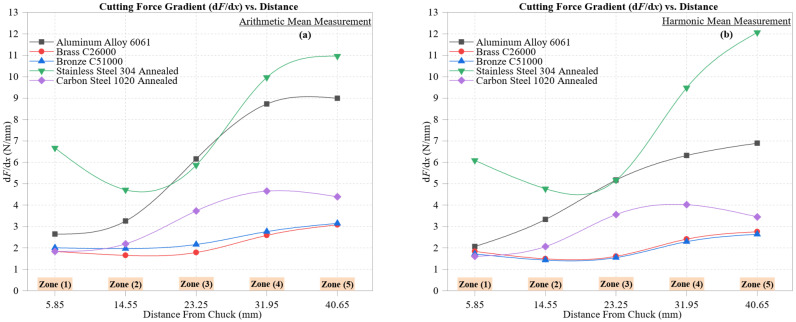
Cutting force gradient (d*F*/d*x*) along machining distance: (**a**) AM and (**b**) HM approaches. Note: Gradients shown represent finite differences (deltas) computed from discrete zone-wise force measurements, as detailed in Equation (3), not continuous derivatives.

Figure 6 shows the cutting force gradient (*dF*/*dx*) for the five materials across the five machining zones, calculated using both arithmetic (Figure 6a) and harmonic (Figure 6b) mean values. These gradients quantify the increase in cutting force per unit distance from the chuck, providing insight into strain hardening, thermal dissipation, and tool–material interaction dynamics during progressive tool engagement.

Stainless Steel 304 Annealed exhibits the steepest gradient, peaking at 10.96 N/mm (AM) and 12.07 N/mm (HM) in Zone 5, reflecting a pronounced escalation in cutting resistance. This behavior is linked to its high strain-hardening rate and low thermal conductivity (~16 W/m·K), which promotes rapid heat buildup and flank wear in distal regions.

Aluminum Alloy 6061 also exhibits high gradients: 8.99 N/mm (AM) and 6.90 N/mm (HM). The AM values more clearly reflect transient spikes from built-up edge (BUE) and thermal softening, while HM smoothing highlights the underlying deformation trend.

Brass C26000 and Bronze C51000 exhibit more linear and stable behavior. The brass ranges from 1.84 to 3.08 N/mm (AM) and 1.83 to 2.76 N/mm (HM), indicating low resistance buildup. The bronze reaches 3.15 N/mm (AM) and 2.64 N/mm (HM), which is slightly higher due to its greater hardness and less ductile chip formation.

Carbon Steel 1020 Annealed falls between the extremes. Its gradient increases from 1.84 to 4.39 N/mm (AM) and 1.61 to 3.45 N/mm (HM) across zones, consistent with moderate work hardening and its ferrite–pearlite microstructure.

Across all materials, harmonic mean gradients are lower in magnitude and less volatile, particularly in the aluminum and stainless steel, demonstrating their ability to suppress zone-specific spikes caused by thermal overload, tool chatter, or BUE events.

Gradient curve shapes confirm that cutting resistance accelerates, rather than increases uniformly, in materials with low conductivity or high strain sensitivity. These results serve as a diagnostic tool to identify zones of instability and thermal–mechanical amplification, offering valuable predictive insight for toolpath optimization.

**Figure 7:**
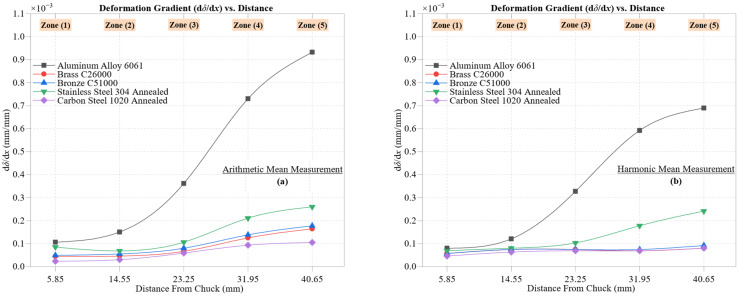
Deformation gradient (d*δ*/d*x*) along machining distance; (**a**) AM and (**b**) HM approaches.

Figure 7 illustrates the deformation gradient ($\frac{d\delta }{dx}$), representing the rate of increase in tool-induced deformation per unit distance from the chuck across five machining zones. This metric captures the spatial evolution of compliance and is essential for evaluating machining stability and the risk of tool deflection during CNC turning.

In the arithmetic mean analysis (Figure 7a), Aluminum Alloy 6061 shows the steepest and most non-linear trend, rising from 1.07 × 10^−4^ mm/mm (Zone 1) to 9.32 × 10^−4^ mm/mm (Zone 5). This reflects localized thermal softening and increased compliance due to low hardness (~95 HBW) and high thermal conductivity (~205 W/m·K), which promote heat-induced yielding as the tool progresses.

Stainless Steel 304 Annealed exhibits a gradual but consistent gradient increase from 8.53 × 10^−5^ to 2.60 × 10^−4^ mm/mm, indicating sustained deformation accumulation resulting from low thermal conductivity and elevated work hardening. Carbon Steel 1020, in contrast, peaks at only 1.05 × 10^−4^ mm/mm, showing lower compliance and higher dimensional stability due to its ferrite–pearlite matrix.

Brass C26000 and Bronze C51000 display moderate and stable gradients. The brass ranges from 4.46 × 10^−5^ to 1.65 × 10^−4^ mm/mm, while the bronze is slightly higher due to its greater strength and frictional resistance. These values reflect effective chip evacuation and reduced thermal distortion in both materials.

Harmonic mean analysis (Figure 7b) shows lower and smoother gradients across all materials. The aluminum still reaches the highest value (6.90 × 10^−4^ mm/mm), but its progression is more gradual than in the AM case. The stainless steel peaks at 2.41 × 10^−4^ mm/mm, reinforcing its tendency for cumulative plastic strain under load.

The carbon steel, brass, and bronze remain tightly grouped and exhibit near-linear harmonic gradients, especially between Zones 3 and 5. This demonstrates the harmonic mean’s robustness in highlighting steady-state behavior and suppressing localized deformation surges, particularly in ductile or structurally stable materials.

Overall, deformation gradient analysis helps identify zones of machining instability and tool deflection. The contrast between AM and HM results reinforces the value of a dual-statistical approach: AM reveals transient spikes, while HM highlights stable trends and filters out variability.

### 4.3. Load-to-Deformation Coupling and Stiffness Characterization

**Figure 8:**
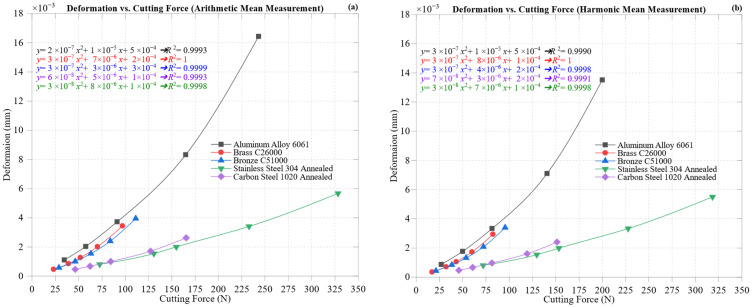
Relationship between deformation and cutting force; (**a**) AM and (**b**) HM measurements.

Figure 8 illustrates the relationship between cutting force (*F*_c_) and maximum deformation (*δ*) across the five materials, using both arithmetic mean (Figure 8a) and harmonic mean (Figure 8b) formulations. The observed quadratic trends confirm the non-linear elastic–plastic response of metals during machining, where deformation is governed not only by mechanical load but also by intrinsic properties like hardness, thermal conductivity, and ductility. Here, ‘stiffness gradient’ denotes the spatial evolution of material stiffness ($\frac{dF}{d\delta }$) across machining zones, reflecting changes in resistance to deformation along the cutting path.

Aluminum Alloy 6061 displays the most prominent non-linear behavior, with deformation increasing from 0.00111 mm at *F*_c_ = 34.61 N to 0.01643 mm at *F*_c_ = 243.08 N in the AM case, and from 0.00086 mm to 0.01352 mm in the HM case. Both curves exhibit near-perfect fits (*R*^2^ = 0.9993 for AM, 0.9990 for HM), highlighting the aluminum’s low hardness and high ductility, which promote plastic strain accumulation as load rises.

Brass C26000 presents a near-linear and highly stable trend, rising from 0.00047 mm at 23.09 N to 0.00345 mm at 97.02 N (AM), and 0.00035 mm to 0.00293 mm (HM), with *R*^2^ = 1.000 in both cases. This exceptional linearity reflects the brass’s fine microstructure and excellent machinability, showing minimal deviation from expected elastic behavior.

Bronze C51000, though slightly harder, follows a more curved trajectory, reaching 0.00395 mm at 111.32 N (AM) and 0.00339 mm at 95.67 N (HM). The models remain highly accurate (*R*^2^ = 0.9999 AM, 0.9998 HM), and the smoother increase suggests moderate plasticity under rising force.

Stainless Steel 304 Annealed shows a moderate increase in δ with force, ranging from 0.00081 mm to 0.00567 mm (AM) as *F*_c_ increases from 72.66 N to 328.23 N, and achieving 0.00550 mm at 318.36 N (HM). The fits are excellent (*R*^2^ = 0.9993 AM, 0.9991 HM), despite the steel’s strain-hardening behavior and austenitic structure, which generally limit deformation.

Carbon Steel 1020 Annealed offers a balanced response between stiffness and compliance, with deformation increasing from 0.00047 mm to 0.00263 mm (AM) and 0.00047 mm to 0.00240 mm (HM). The regression fits (*R*^2^ = 0.9998 for both) indicate highly predictable stiffness performance under load. 

This gradual evolution in deformation for both the stainless steel and carbon steel may stem from dynamic grain refinement and progressive strain hardening near the surface during machining [43], which alters their mechanical resistance along the cut.

Together, the regression models in both plots confirm that deformation response is governed by material-specific microstructural traits, such as phase composition, grain size, and thermal conductivity. The contrast between the rapid deformation of Aluminum 6061 and the stable response of the brass and carbon steel highlights critical differences in machinability relevant to high-precision applications.

The small differences between AM and HM curves further validate the dataset’s consistency, with both approaches supporting robust machinability assessments.

**Figure 9:**
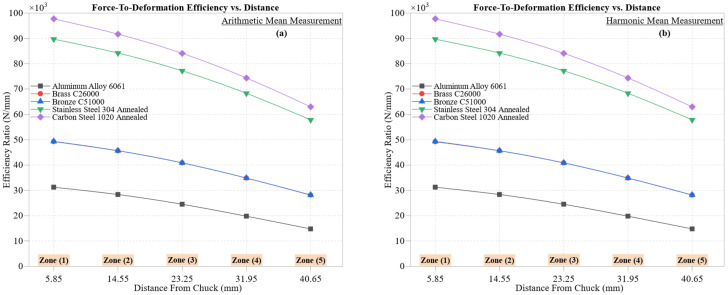
Force-to-Deformation Efficiency Along Machining Distance (**a**) AM and (**b**) HM Approaches.

Figure 9 presents the cutting force-to-deformation efficiency ($\eta =\frac{F_{c}}{\delta }$) across the five machining zones for all materials, computed using both arithmetic (Figure 9a) and harmonic (Figure 9b) mean formulations. This metric reflects the material’s capacity to convert applied force into effective mechanical output while limiting elastic–plastic displacement, serving as a proxy for energy efficiency, stiffness, and dimensional resilience during machining.

Carbon Steel 1020 Annealed consistently ranks highest in efficiency, with *η* declining from ~97,750 N/mm in Zone 1 to ~63,000 N/mm in Zone 5. This gradual decay underscores the material’s structural stability, with minimal thermal softening due to moderate thermal conductivity (~51 W/m·K). Its ability to maintain high resistance to deformation even in distal regions supports its suitability for dimensionally critical components.

Stainless Steel 304 Annealed exhibits a similar descending profile (from ~89,695 N/mm to ~57,850 N/mm), but with a steeper drop. The material’s low thermal conductivity (~16 W/m·K) exacerbates localized heat buildup, reducing stiffness and increasing deformation susceptibility in Zones 4–5. These effects increase the likelihood of flank wear and surface irregularities, thereby limiting the machining consistency of stainless steel under dry conditions.

Aluminum Alloy 6061 records the lowest efficiency across all zones, starting at ~31,244 N/mm and decreasing to ~14,791 N/mm. Despite its high thermal conductivity (~205 W/m·K), its low hardness (~95 HBW) and ductility result in pronounced plastic deformation. This makes the aluminum particularly prone to built-up edge (BUE) formation, chatter, and surface smearing, especially in unsupported cutting zones where vibrational sensitivity increases.

Brass C26000 and Bronze C51000 show overlapping trends, with *η* dropping from ~49,200 N/mm in Zone 1 to ~28,150 N/mm in Zone 5 for both. These values reflect their comparable hardness and copper-based metallurgy, which aid heat dissipation and reduce friction. Nonetheless, the efficiency decline with tool advancement hints at increased chip adhesion or secondary shear zone instability.

The harmonic mean plots (Figure 9b) produce lower numerical values across materials due to their sensitivity to small deformation spikes. This provides a conservative estimate that is particularly effective at capturing transient compliance events, such as tool vibrations or chatter, that may be underrepresented in arithmetic mean evaluations. Despite these differences, material efficiency rankings remain consistent between both statistical approaches, reinforcing the robustness of *η* as a comparative performance metric.

From a machining optimization perspective, the observed decline in zone-wise efficiency highlights the growing influence of thermomechanical instability with increasing tool distance from the chuck. This suggests that strategies such as zonal feed modulation, adaptive cooling, or gradient toolpaths may enhance stability and extend tool life. The superior performance of the carbon steel and brass in retaining high *η* further supports their selection for precision, energy-efficient CNC turning, especially where dimensional control is critical.

In conclusion, the force-to-deformation efficiency metric not only captures energy transfer fidelity but also reflects thermomechanical durability and form accuracy. Its dual statistical representation adds depth to material machinability assessment and enables predictive modeling for process improvement.

**Figure 10:**
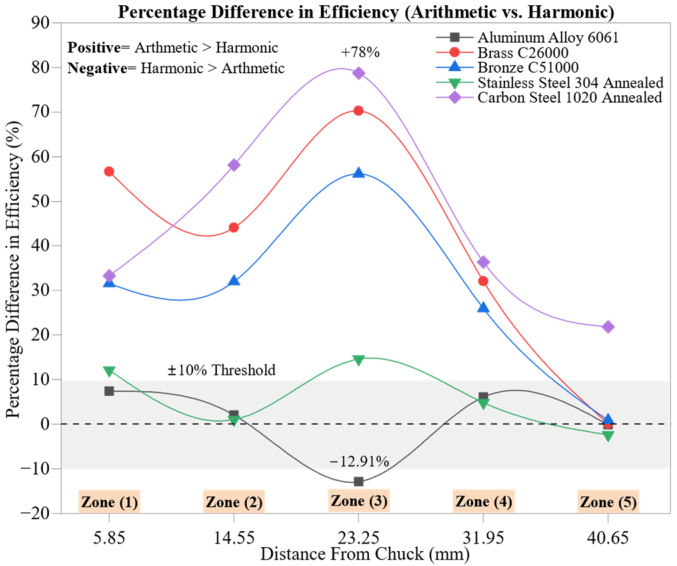
Percentage difference in force-to-deformation efficiency between AM and HM methods across machining zones.

Figure 10 illustrates the percentage difference in force-to-deformation efficiency (*η*) between arithmetic mean (AM) and harmonic mean (HM) formulations across five machining zones for all materials. This comparative analysis reveals how statistical averaging affects perceived machining efficiency, particularly under transient tool–material interaction conditions.

The most prominent differences appear at Zone 3 (23.25 mm), where Carbon Steel 1020 Annealed shows the highest deviation at +78.72%, followed by Brass C26000 (+70.26%), Bronze C51000 (+56.10%), and Stainless Steel 304 Annealed (+14.58%). These values indicate that AM values in this region are disproportionately influenced by local anomalies, such as force peaks or minimal deformation readings, that skew the average upward, amplifying apparent efficiency. This effect is particularly pronounced in materials with rigid microstructures, where mid-span dynamics may introduce irregular tool–workpiece interactions or transitional wear behavior.

Aluminum Alloy 6061 diverges from this trend, showing a negative deviation (−12.91%) at Zone 3. This suggests that HM produces a higher efficiency estimate than AM in this case. The discrepancy likely arises from the aluminum’s low hardness, high ductility, and thermal conductivity (~205 W/m·K), which result in elevated plastic deformation that disproportionately affects AM values. The harmonic mean, less influenced by extreme values, offers a more robust estimate in such ductile systems.

Early zones (Zones 1 and 2) also demonstrate considerable deviations, especially for the brass (+56.63%, +44.08%) and carbon steel (+33.30%, +58.10%). These differences reflect the mechanical instability and tool adaptation that dominate the initial stages of cutting, where variations in engagement conditions and cutting depth can strongly affect force and deformation outputs.

In contrast, the final zone (Zone 5, 40.65 mm) shows convergence between AM and HM estimates for all materials. The differences narrow to below ±2.5%, e.g., the aluminum (−0.16%), brass (+0.10%), bronze (+0.85%), and stainless steel (−2.38%), indicating the emergence of quasi-steady-state cutting. This alignment confirms that machining behavior becomes more consistent and statistically stable as tool–material interaction progresses outward and transient anomalies diminish.

This sensitivity analysis highlights the importance of dual statistical treatment in machining studies. While the AM captures process peaks and energetic outliers, the HM offers a smoothed profile that is less biased by extreme fluctuations. Their divergence, especially in mid-span zones, provides insight into localized thermomechanical instability, while their convergence at distal zones reflects process stabilization.

Ultimately, incorporating both statistical perspectives enhances diagnostic depth in machining research and supports more accurate assessment of zone-specific efficiency, material compliance, and predictive control strategies for adaptive toolpath planning.

**Figure 11:**
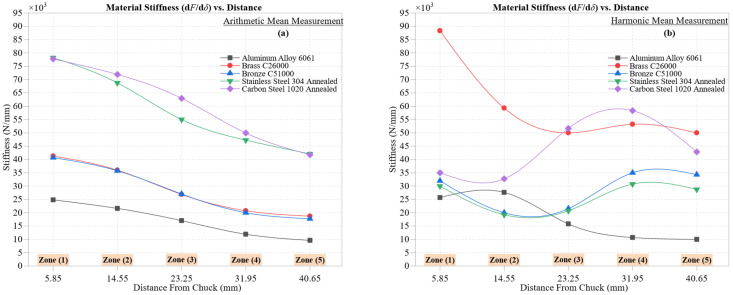
Material stiffness (d*F*/d*δ*) along machining distance; (**a**) AM and (**b**) HM approaches.

Figure 11 presents the evolution of material stiffness, defined as the ratio $\frac{dF}{d\delta }$ across five machining zones for all materials using arithmetic mean (Figure 11a) and harmonic mean (Figure 11b) formulations. This metric reflects each material’s capacity to resist elastic–plastic deformation under increasing cutting loads, directly influencing dimensional stability and energy absorption during CNC turning.

In the arithmetic mean analysis, stiffness consistently decreases with radial distance from the chuck, reflecting cumulative effects of thermal softening, strain localization, and tool wear. Stainless Steel 304 Annealed maintains the highest stiffness values, falling from 78,200 N/mm in Zone 1 to 42,111 N/mm in Zone 5. This robustness stems from its austenitic structure and high strain-hardening capacity, although the decline is attributed to heat buildup due to its low thermal conductivity (~16 W/m·K).

Carbon Steel 1020 Annealed shows a similar trend, decreasing from 77,752 N/mm to 41,749 N/mm, supported by its ferrite–pearlite microstructure and moderate thermal conductivity (~51 W/m·K). The drop across zones highlights increased tool–material friction and potential flank wear, leading to tool deflection and reduced machining precision.

Aluminum 6061 exhibits the lowest stiffness values, descending sharply from 24,864 N/mm to 9647 N/mm. Despite its high thermal conductivity (~205 W/m·K), its low hardness and high ductility result in significant elastic–plastic deformation under even moderate loads. The presence of built-up edge (BUE) further exacerbates dimensional instability, especially in unsupported regions.

Brass C26000 and Bronze C51000 exhibit intermediate stiffness profiles, with the brass decreasing from 41,271 N/mm to 18,726 N/mm and the bronze from 40,714 N/mm to 17,732 N/mm across the machining zones. Their similar stiffness degradation reflects comparable thermal conductivity and moderate mechanical strength. However, subtle deviations arise due to differences in microstructural characteristics: the brass is primarily a single-phase α alloy, which promotes uniform plastic flow, whereas the bronze often exhibits a dual-phase *α*–*β* structure, where the more challenging *β* phase introduces localized resistance and strain heterogeneity during chip formation.

The harmonic mean analysis offers a refined perspective by attenuating extreme deformation spikes. Here, Stainless Steel 304 leads with higher and more stable values, ranging from 88,333 N/mm to 50,000 N/mm, emphasizing its sustained rigidity under variable loads.

Interestingly, Carbon Steel 1020 exhibits reduced variability in harmonic stiffness (35,000–42,857 N/mm), implying consistent mechanical response and minimal susceptibility to localized deflection. This contrasts with the broader trend in arithmetic data, reaffirming the harmonic mean’s robustness against transient deformation noise.

A notable observation emerges in the behavior of the brass and bronze: while the bronze is stiffer under arithmetic analysis, the brass overtakes it in harmonic stiffness in later zones. This reversal suggests that the bronze may experience more frequent soft deformation episodes (potentially due to higher tin content), which the harmonic mean effectively penalizes, demonstrating the method’s sensitivity to subtle compliance events.

For Aluminum 6061, harmonic stiffness values remain marginally higher than their arithmetic counterparts, with a slower decline. This indicates fewer deformation outliers, albeit confirming persistent plastic energy loss and reduced structural resistance under load.

In summary, the dual statistical approaches offer complementary insights: the arithmetic mean captures peak effects and macro-level degradation trends, while the harmonic mean reveals consistent material performance, free from the influence of outliers. Together, they provide a comprehensive framework for assessing stiffness degradation, thermal–mechanical instability, and tool–material interaction dynamics, all of which are critical to optimizing machining parameters and enhancing precision across varying cutting conditions.

**Figure 12:**
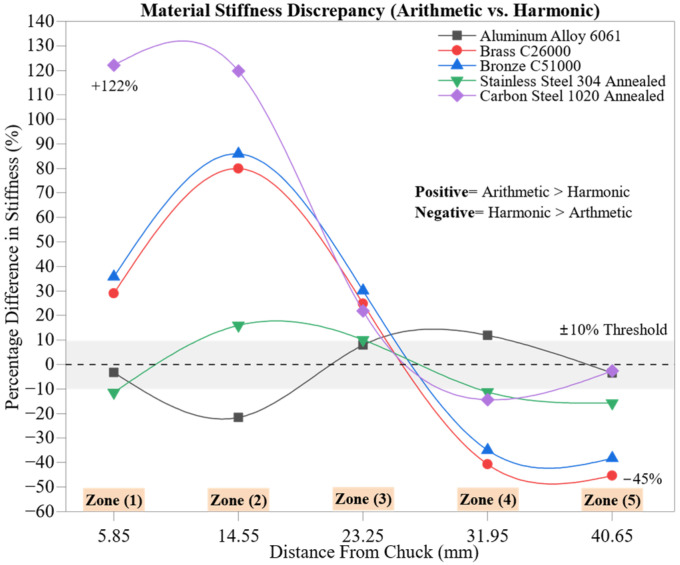
Percentage difference in material stiffness between AM and HM methods across machining zones.

Figure 12 illustrates the percentage differences in stiffness values ($\frac{dF}{d\delta }$) obtained via arithmetic mean (AM) and harmonic mean (HM) formulations across the five machining zones. Positive deviations indicate that the arithmetic mean yielded higher stiffness values, while negative deviations reflect dominance by the harmonic mean. This comparison reveals how the chosen averaging method impacts perceived material rigidity, especially under varying deformation regimes.

In Zone 1 (5.85 mm), significant overestimations of stiffness by the arithmetic mean are observed for Carbon Steel 1020 Annealed (+122.1%), Bronze C51000 (+35.7%), and Brass C26000 (+29.0%). These early-zone discrepancies likely stem from the sensitivity of arithmetic means to initial deformation variability, especially under transient tool engagement. In contrast, Aluminum 6061 (−3.3%) and Stainless Steel 304 Annealed (−11.5%) show negative values, indicating early-stage alignment between AM and HM methods—likely due to their consistent or smoothly varying deformation responses.

As the cutting progresses into Zone 2 (14.55 mm), the percentage differences for the brass (+79.9%), bronze (+85.9%), and carbon steel (+119.7%) increase, reinforcing the notion that the arithmetic mean exaggerates stiffness as force and deformation escalate. Simultaneously, Aluminum 6061 shows a sharp swing to −21.7%, while Stainless Steel 304 transitions into a modest positive deviation (+16.0%), suggesting a localized shift in force–deformation balance and microstructural response.

In Zone 3 (23.25 mm), the differences begin to stabilize. The brass, bronze, and carbon steel maintain moderate positive values (+24.8%, +30.1%, and +21.9%, respectively), indicating a residual influence of arithmetic averaging. Meanwhile, Aluminum 6061 reverses its prior trend and displays a mild positive deviation (+7.9%), possibly reflecting more consistent mechanical behavior. Stainless Steel 304 remains closely aligned across both formulations (+10.0%), underscoring its relatively stable deformation resistance.

Zone 4 (31.95 mm) marks a critical turning point, with all materials except the aluminum now showing negative deviations, signaling the emergence of harmonic mean dominance. Notably, the brass (−40.8%) and bronze (−34.9%) shift to substantially below zero, suggesting that the harmonic mean more accurately captures small but increasing compliance. The stainless steel (−7.6%) and carbon steel (−5.5%) also exhibit negative trends, consistent with the increasing tool–material thermal interaction. The aluminum retains a slight positive offset (+11.9%), albeit reduced from earlier zones.

In the final zone, Zone 5 (40.65 mm), harmonic mean superiority becomes fully apparent for most materials. The brass and bronze exhibit their most significant negative deviations (−45.4% and −38.3%, respectively), while Stainless Steel 304 (−15.8%) and Carbon Steel 1020 (−2.6%) continue to converge toward harmonic values, reflecting stabilized cutting conditions. Aluminum 6061 returns to a mild negative differential (−3.5%), suggesting reduced influence from outliers and localized deformation spikes.

This analysis emphasizes the statistical sensitivity of stiffness metrics and highlights the limitations of arithmetic averaging under non-linear, transient, or thermally unstable machining conditions. Arithmetic means often inflate stiffness when deformation distributions are skewed or affected by sporadic compliance events, which is particularly evident in early zones. In contrast, harmonic means offer superior robustness in late zones, where steady-state cutting allows for more consistent deformation profiles.

Together, these findings reinforce the importance of dual-statistical treatments in machining research. Relying solely on arithmetic means can misrepresent material rigidity, especially for alloys with pronounced ductility or variable thermal response. The inclusion of harmonic means ensures a more nuanced and resilient characterization of stiffness, which is crucial for process optimization, toolpath planning, and material selection in precision machining.

To provide a consolidated overview of the machining behavior across the studied materials, Table 2 presents a summary of key trends in cutting force, deformation, force-to-deformation efficiency, and stiffness variation across the five machining zones. This summary complements the detailed analyses in the preceding sections and supports the multivariate comparisons presented in Section 4.4.

### 4.4. Multivariate Behavior and Material Clustering

**Figure 13:**
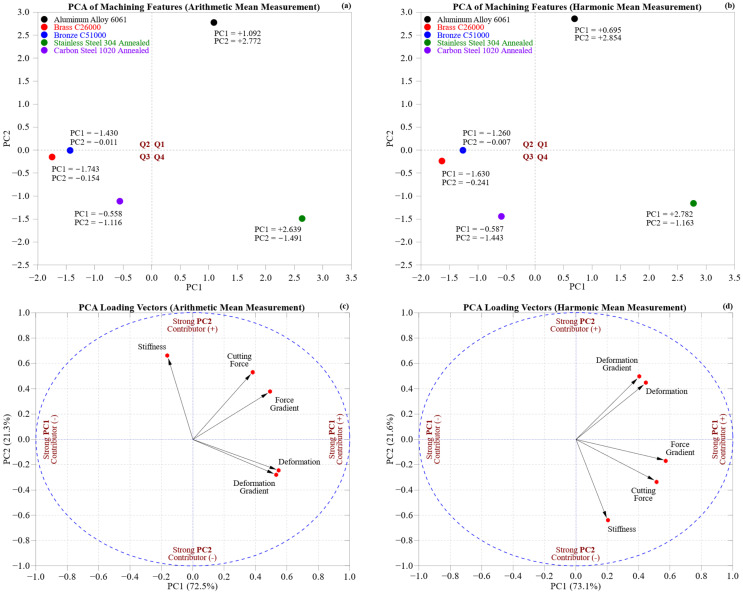
Principal component analysis (PCA) of machining features: (**a**) AM and (**b**) HM measurements; (**c**) PCA loading vectors for AM with unit circle; (**d**) PCA loading vectors for HM with unit circle.

Figure 13 demonstrates the multivariate machining characteristics of the five materials through principal component analysis (PCA) applied to arithmetic mean (AM) and harmonic mean (HM) datasets. Biplots (Figure 13a,b) reveal material clustering, and loading vectors (Figure 13c,d) showcase the key variables. The first two principal components account for 93.8% (AM) and 94.7% (HM) of the total variance, which verifies the success of dimensionality reduction.

Table 3 shows that PC1 is predominantly influenced by deformation, deformation gradient, and force gradient, which together explain 82.7% of its variance in the AM and 69.3% in the HM. PC2, on the other hand, is mainly determined by stiffness and cutting force, making up 71.8% of the variance in the AM and 52.1% in the HM. These trends provide a consistent physical interpretation across both statistical methods.

In the AM biplot (Figure 13a), Stainless Steel 304 Annealed is prominently situated along PC2 (PC1 = +2.639, PC2 = −1.491), demonstrating its stiffness and high cutting force. Aluminum 6061 is found in the upper-right quadrant (PC1 = +1.092, PC2 = +2.772), known for its ductility and deformation characteristics. Brass C26000 and Bronze C51000 group together in the negative PC1 area (the brass: −1.743, −0.154; the bronze: −1.430, −0.007), revealing comparable compliance profiles. Carbon Steel 1020 Annealed is located close to the origin (PC1 = −0.558, PC2 = −1.116), indicating a well-balanced machining response.

The HM biplot (Figure 13b) reinforces these findings. Stainless Steel 304 is still a key player (PC1 = +2.782, PC2 = −1.163), and Aluminum 6061 persists with its deformation-related characteristics (PC1 = +0.695, PC2 = +2.854). The brass and bronze continue to form their cluster, while Carbon Steel 1020 remains close to the center (PC1 = −0.587, PC2 = −1.443).

Quadrant analysis enhances interpretative clarity: Q1 (PC1 > 0, PC2 > 0) features Aluminum 6061, associated with high compliance and efficiency. Q3 (PC1 < 0, PC2 < 0) comprises Carbon Steel 1020, reflecting moderate performance. Q4 (PC1 > 0, PC2 < 0) contains Stainless Steel 304, characterized by stiffness and substantial cutting force. Q2 is currently empty.

Loading the vector plots (Figure 13c,d) strengthens these findings. In both the AM and HM, PC1 focuses on features related to deformation, while PC2 highlights stiffness and cutting force. Nonetheless, AM showcases large-scale deformation impacts, whereas the HM more effectively captures small variations, uncovering compliance differences in materials such as bronze and carbon steel. In the HM, stiffness transitions to PC1, and deformation-related variables become more dispersed, emphasizing localized mechanical sensitivity.

Force and deformation gradients, which are sensitive to zone-wise variations, appear more distinct in the HM, thereby improving inter-zone differentiation. The near-opposite loading patterns between the AM and HM reflect complementary perspectives: the AM captures broad deformation trends, while the HM focuses on fine-scale mechanical responses.

AM and HM PCAs provide a comprehensive perspective on machining behavior, encompassing both macro-scale deformation and micro-scale sensitivity. This dual view is crucial for enhancing material selection, toolpath planning, and cutting strategies tailored to specific zones in CNC applications.

### 4.5. Composite Machinability Evaluation

**Figure 14:**
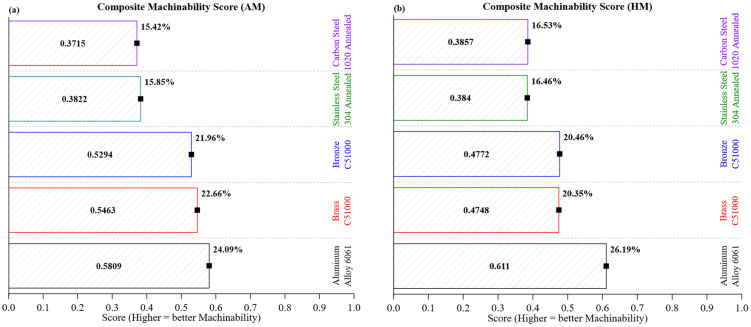
Composite machinability score by material: (**a**) AM and (**b**) HM evaluations.

Figure 14 displays the composite machinability ranking of the five materials tested based on the composite machinability score (CMS). This metric combines four key machining attributes: cutting force, deformation, stiffness, and force-to-deformation efficiency. To enhance robustness against data skewness and variations in magnitude, the CMS is calculated using both the arithmetic mean (Figure 14a) and the harmonic mean (Figure 14b).

According to the arithmetic mean, Aluminum Alloy 6061 leads with a CMS of +0.5809, thanks to its low cutting force, high compliance, and efficient heat dissipation (~167 W/m·K), though it has low hardness (~95 HBW). Brass C26000 ranks next at +0.5463, followed closely by Bronze C51000 at +0.5294, both showcasing effective chip formation, favorable energy profiles, and moderate thermal properties. Stainless Steel 304 Annealed and Carbon Steel 1020 Annealed have lower scores of +0.3822 and +0.3715, respectively, reflecting their increased force requirements and less effective thermal management.

The harmonic mean analysis highlights consistency while penalizing variability, supporting these trends. The CMS for Aluminum 6061 rises to +0.6110, reinforcing its machinability benefits in stable environments. Bronze C51000 at +0.4772 slightly surpasses Brass C26000 at +0.4748, with their relative standings shifting slightly due to the sensitivity of harmonic averaging to micro-instabilities. Meanwhile, Stainless Steel 304 and Carbon Steel 1020 show minor improvements to +0.3840 and +0.3857, respectively, but they still rank lower because of ongoing inefficiencies.

These consistent rankings confirm the statistical resilience and physical interpretability of the CMS framework. Specifically, materials that are low in hardness but high in thermal conductivity, like aluminum and brass, consistently perform better than their stiffer, low-conductivity alternatives, such as stainless steel. The dual-statistical approach effectively captures both average behavior and response stability, providing a reliable tool for assessing machining performance.

To the author’s knowledge, this is the first implementation of a dual-statistical CMS framework that includes zone-specific stiffness and efficiency descriptors. The CMS findings highlight that machinability is influenced by the interaction of mechanical compliance, thermal behavior, and energetic efficiency. This detailed index supports informed decision-making in material selection, process planning, and tool life enhancement in CNC turning settings.

## 5. Conclusions

This study developed a statistically grounded and physically insightful framework for evaluating the zone-wise machinability of five industrial-grade engineering materials: Aluminum Alloy 6061, Brass C26000, Bronze C51000, Stainless Steel 304 Annealed, and Carbon Steel 1020 Annealed, under dry CNC turning conditions. Employing both arithmetic and harmonic mean formulations, the methodology enabled a dual-mode assessment of central trends in cutting force and deformation, while also extending the analysis to higher-order descriptors, including force gradients, deformation rates, stiffness, and energy efficiency.

The cutting force results revealed clear material-specific patterns across the machining zones. Stainless Steel 304 exhibited the highest resistance, peaking at 328 N in Zone 5, due to pronounced strain hardening and poor thermal dissipation. In contrast, Brass C26000 demonstrated smooth force transitions across zones, affirming its stable machinability. Deformation results echoed these trends: Aluminum 6061 showed the largest displacement (0.0164 mm), reflecting its ductility, whereas Carbon Steel 1020 maintained minimal deformation (<0.0027 mm), indicative of high rigidity and mechanical resistance.

The gradient analysis uncovered spatial dynamics in machining behavior. Cutting force gradients ($\frac{dF}{dx}$) identified zones of abrupt force variation, while deformation gradients ($\frac{d\delta }{dx}$) exposed areas of local compliance and material softening. The derived stiffness metric ($\frac{dF}{d\delta }$) declined progressively across zones, particularly in Stainless Steel 304, highlighting the cumulative effects of thermal and tool wear with increased cutting distance.

A novel force-to-deformation efficiency ratio provided further insight into energetic performance. Materials like Carbon Steel 1020 and Brass C26000 exhibited high efficiency values (>97,000 N/mm in Zone 1), confirming their capacity to resist deformation while maintaining stable cutting conditions, which is critical for dry-machining applications.

The principal component analysis (PCA) effectively reduced dimensional complexity and revealed distinct material clusters. Stainless Steel 304 and Aluminum 6061 occupied opposite PCA quadrants, representing extremes of hardness and ductility, respectively. The brass and bronze formed a central cluster, while Carbon Steel 1020 was positioned near the origin, underscoring its balanced machining profile. The PCA quadrant framework (Q1–Q4) further classifies materials based on dominant behaviors, with stiffness in Q4 and deformation resilience in Q1, thereby linking mechanical response to process characteristics.

To synthesize the results, a composite machinability score (CMS) was established by integrating normalized metrics for cutting force, deformation, stiffness, and efficiency of force-to-deformation into a single index. Aluminum Alloy 6061 achieved the highest CMS value at +0.5809, closely followed by Brass C26000 at +0.5463 and Bronze C51000 at +0.5294. In contrast, Stainless Steel 304 Annealed and Carbon Steel 1020 Annealed ranked lower, scoring +0.3822 and +0.3715, respectively, owing to their increased cutting forces and reduced energy efficiency. The harmonic mean analysis preserved the overall ranking, with Aluminum 6061 improving its CMS to +0.6110. Notably, Carbon Steel 1020 slightly outperformed Stainless Steel 304 in this analysis, recording CMS values of +0.3857 and +0.3840, respectively. This consistent ranking of materials across both statistical approaches underscores the robustness and dependability of the CMS framework in evaluating machinability performance among various material categories.

In summary, this study presents a comprehensive and scalable framework for assessing machinability, integrating experimental force–deformation data with derivative mechanical metrics and dual-statistical modeling. The proposed methodology enables informed decision-making in material selection, toolpath planning, and process optimization, promoting energy-efficient and precision-driven manufacturing strategies across a wide range of engineering alloys. To the best of the author’s knowledge, this is the first study to unify zone-wise gradient-based mechanical descriptors with dual-statistical analysis and PCA clustering to construct a composite machinability score across multiple engineering materials.

## Figures and Tables

**Table 1:** Key mechanical and thermal properties of the investigated materials.

Material	Yield Stress (MPa)	Ultimate Tensile Strength (MPa)	Hardness (HBW)	Thermal Conductivity (W/m·K)	Density (kg/m^3^)	Elastic Modulus (GPa)
Aluminum Alloy 6061	276	310–350	95	205	2700	69
Brass C26000	~138	~310	~70–80	109	8530	~100–110
Bronze C51000	241	414	~110	~53	8800	117
Stainless Steel 304 Annealed	215	505	~150	16	8000	193
Carbon Steel 1020 Annealed	250	395–435	140	51	7870	205

**Table 2:** Summary of cutting force, deformation, efficiency, and stiffness trends, and PCA clustering across materials and zones.

Material	Maximum Cutting Force (N)	Maximum Deformation (mm)	Force-to-Deformation Efficiency (N/mm) Zone 1 → Zone 5	Stiffness Decline (%)	PCA Cluster Position
Aluminum 6061	243	0.01640	31,244 → 14,791	High decline	High compliance
Brass C26000	97	0.00345	49,226 → 28,152	Moderate decline	Stable
Bronze C51000	111	0.00395	49,325 → 28,178	Moderate decline	Stable
Carbon Steel 1020 Annealed	165	0.00263	97,750 → 63,000	Low decline	High stiffness
Stainless Steel 304 Annealed	328	0.00567	89,695 → 57,850	Low decline	High stiffness

**Table 3:** PCA loading matrices and variable contributions (%) for arithmetic mean (AM) and harmonic mean (HM) cases. All loadings are normalized and dimensionless.

Variable	PC1 (AM)		PC2 (AM)		PC1 (HM)		PC2 (HM)	
	Loading	Contribution (%)	Loading	Contribution (%)	Loading	Contribution (%)	Loading	Contribution (%)
Cutting Force	+0.3831	17.5	+0.5300	28.1	+0.5157	26.6	−0.3364	11.3
Deformation	+0.5478	30.0	−0.2450	6.0	+0.4467	20.0	+0.4491	20.2
Stiffness	−0.1613	2.6	+0.6613	43.7	+0.2051	4.2	−0.6387	40.8
Force Gradient	+0.4931	24.3	+0.3789	14.4	+0.5733	32.9	−0.1704	2.9
Deformation Gradient	+0.5329	28.4	−0.2796	7.8	+0.4047	16.4	+0.4982	24.8

## Data Availability

The data presented in this study are available on request from the corresponding author due to privacy and legal reasons.

## Abbreviations

The following abbreviations are used in this manuscript:

CNC	computer numerical control
PCA	principal component analysis
CMS	composite machinability score
AM	arithmetic mean
HM	harmonic mean
BUE	built-up edge
HBW	Brinell hardness (according to ISO 6506)
PC1, PC2	principal components 1, 2
IQR	interquartile range
SD	standard deviation
*R*^2^	coefficient of determination
ISO	International Organization for Standardization
ISO 3685	Tool-life testing with single-point turning tools
ISO 513	Classification and application of hard cutting materials
ISO 1832	Indexable inserts for cutting tools—designation
